# Supplementary material for: Methods and considerations for estimating parameters in biophysically detailed neural models with simulation based inference
Source: PLoS Comput Biol. 2024 Feb 26;20(2):e1011108. doi: 10.1371/journal.pcbi.1011108 (PMC10919875; doi:10.1371/journal.pcbi.1011108)
Supplement: Attachment — Submitted filename: hnn_sbi_reviewer_response.pdf [file pcbi.1011108.s001.pdf]

<u>Response color key</u>

Original reviewer comment

Author response

Revised manuscript text

Original manuscript text

Reviewer #1:

This ms focuses on techniques for parameter inference of biophysically detailed models. This is challenging due to the high dimensionality of the parameter space. The ms attempts to address these challenges and offers an interesting overview of related work and literature. Below I am including some comments and suggestions for various parts of the ms.

Thank you for the thorough review and very helpful feedback!

<u>Introduction</u>

It is not clear to me how the "limited set of parameters …chosen"(l. 17) are chosen? If the current approach aims at overcoming the high number of parameters in detailed models (l.14), this is not achieved by picking a few parameters and fixing others as it is done in the Results, see also below.

This is a very good remark and we agree it is not clear in the manuscript. Indeed, a major limitation of amortized posterior estimation with SBI is the need to restrict the parameters allowed to vary. Our intention with this section is to emphasize that clear hypotheses formulated in terms of a small set of model parameters are a necessity to perform amortized inference. We have made the following modifications to clarify this point:

"Then, a limited set of parameters are chosen as the target for estimation. *This limited set is chosen based on a prior hypothesis that a certain set of unknown parameters can be estimated based on data features.* Even with this limited set, the parameter estimation process is complex. The problem is confounded by the fact that there may be many parameter configurations that produce an equally good representation of the data \cite{marder_variability_2006}. *In this study, we focus on the latter problem of identifying parameter indeterminacies in a limited set of parameters, as to date there is no means to estimate large numbers of parameters at once."*

DCM does not assume that parameters are independent as seemingly suggested on l.36 Interactions are described using the posterior correlation matrix, see e.g. K. E. Stephan, N. Weiskopf, P. M. Drysdale, P. A. Robinson, and K. J. Friston, "Comparing hemodynamic

models with DCM.," Neuroimage, vol. 38, no. 3, pp. 387–401, Nov. 2007. There is a mean field factorization over parameters and hyperparameters, not parameters, in the Variational Laplace inference scheme. Only one DCM variant, the so called mean field models (MFM), not others, assume that separate ensembles are coupled by mean field effects, see Marreiros, A. C., Kiebel, S. J., & Friston, K. J. (2010). A dynamic causal model study of neuronal population dynamics. Neuroimage, 51(1), 91-101. Similar remarks apply to comments in Discussion (see also below).

Thank you for your detailed explanation and references provided. As indicated, our comments were based on a misunderstanding of how the mean field assumption was applied, and the lack of posterior covariance analysis in DCM literature (we appreciate you sending along references in which this is done explicitly).

We have removed the statements suggesting this on l.36, and as outlined in responses to comments below.

On a different matter, the lack of validation with empirical data is a significant limitation of the current ms.

This is an important critique and we agree that a lack of comparison to real data was a major limitation. To directly address this concern, we updated the "Beta Event" example in Figure 7 to provide a proof of concept of inference on real data, from a prior published study [Sherman et al., 2016]. Here, we examine averaged Beta Events from two subjects that have different magnitudes. Please note that these new examples required modification of the prior distribution to a range in which the simulated waveforms sufficiently matched the empirical data, which have larger amplitudes than the simulated only waveforms considered in the original text.

In these examples, the proximal variance is the dominant factor controlling the Beta Event amplitude, such that a smaller variance creates a smaller amplitude response. This is due to the fact that, with a small proximal variance, the strong upward current flow counteracts the downward current flow generated by the distal drive to produce an overall smaller downward deflection. As we outline in the text, this proof-of-concept example based on only two subjects is not a thorough investigation making strong scientific predictions on parameters controlling Beta Event amplitude. It nevertheless demonstrates how the HNN-SBI framework can be used based on real data.

Even when using simulated data, like it is done here, one needs to consider noise in the observations (I could not find such reference in the ms). Even if this was used, it is not clear if it related to real noise. Various reasons could account for indeterminacy and lack of parameter identifiability of the sort discussed here, including low SNR or not enough data. This is normally taken care of by carefully parameterising observation noise (besides neural noise) and using real data to assess the predictive validity of models, i.e. whether inferences from one group predict responses of another. Also, different modalities, like LFP vs MEG will have different SNR and observation noise and the sensitivity of predicted and real data on biophysical parameters will depend on both

parameters and observations. In brief, identifiability cannot only be assessed using simulations.

We definitely agree with this critique, unfortunately the role that observation noise plays, and more generally the problem of model misspecification, is largely an open question with SBI techniques and outside the scope of this paper. This is mainly due to a lack of theoretical guarantees on the relationship between observation noise and posterior estimates when neural networks are used for density estimation see:

Ward, D., Cannon, P., Beaumont, M., Fasiolo, M., & Schmon, S. M. (2022, October 12). Robust Neural Posterior Estimation and Statistical Model Criticism. arXiv.Org. https://arxiv.org/abs/2210.06564v1

Cranmer, K., Brehmer, J., & Louppe, G. (2020). The frontier of simulation-based inference. Proceedings of the National Academy of Sciences, 117(48), 30055–30062. https://doi.org/10.1073/pnas.1912789117

Nevertheless this is an important aspect for people to be aware of and have added the following text to the Discussion to highlight that this line of research would be a significant methodological advancement:

"An important limitation of the current study is a characterization of how observation noise impacts posterior parameter inferences, and which summary statistics are more or less robust to such noise. In our study, \PCAthirty{} was found to perform best for parameter recovery, however the information gained from such low variance features may be highly sensitive to noise. Similarly, the hand-crafted summary statistics like \Peak{} and \BandPower{} may be particularly impacted by noise. The sensitivity of neural density estimators to observation noise, and more generally identifying model misspecification, is an open problem in the SBI field \cite{ward_robust_2022, cranmer_frontier_2020} and an important direction for future research. Careful parameterization of observation noise, and establishment of predictive validity of the model using real data, will be critical next steps for robust inferences with the HNN-SBI framework."

Methods

Could be expanded by moving material from elsewhere, see below.

Results

Again, it is not clear how this approach overcomes "challenges of…Bayesian inference" (l. 162) since only few parameters are estimated.

Large parameter spaces are indeed one challenge of inference in detailed models, however, the challenge emphasized in this manuscript is *"estimation of complex posterior distributions that exhibit parameter interactions."* The more pressing challenge with these models is their lack of

access to a likelihood function. The estimation of the posterior distribution with older techniques (namely ABC) were insufficient even for a small number of parameters.

Here we focus on the situation of amortized inference where only a few parameters can be estimated, at the benefit of having a single trained neural network which provides near instantaneous predictions for different observations. We provide a discussion of alternative approaches (i.e. sequential neural posterior estimation (SNPE)) which do enable simultaneous estimation of many parameters, at the expense of your posterior approximations being valid for a single observation (see: *"Comparison to other biophysically detailed neural modeling studies and estimation techniques"*).

Also, recovering original parameter estimates, known as face validity is discussed in many DCM papers. Thus, this criticism appears a bit misleading.

Thank you for pointing this out, we have added the following text early in the manuscript to establish that parameter recovery error is a measure of face validity that has been discussed in previous DCM papers:

"...users can calculate the dispersion of the posterior around this ground truth using parameter recovery error (PRE). This is known as face validity when using simulated data to check if the ground truth parameters can be recovered \cite{kiebel_dynamic_2007, stephan_ten_2010"

Similarly, l. 295 and l. 574 should be toned down. DCM e.g. uses log normal priors, posterior correlations and a complexity term in the Free Energy alongside BMR (see also below) to describe interactions.

We have revised the indicated sections as follows:

~~Additionally, mean field variational inference assumes that model parameters are independent, preventing the ability to capture biologically meaningful parameter interactions.~~

"This example highlights that even with simple simulators, indeterminacies can easily arise necessitating the use of flexible posterior approximators (like masked autoregressive flows) ~~compared to mean field variational inference which cannot handle parameter interactions.~~"

"…this additional complexity can lead to parameter estimation indeterminacy that indicates a compensatory interaction between parameters. ~~Importantly, such parameter interactions would not be revealed with other estimation methods such as mean field variational inference given their assumption of Gaussian and independent parameter distributions (i.e. the Laplace approximation).~~"

It is not clear to me how indeterminacies of parameters are mitigated or addressed here.

Our goal is not to mitigate indeterminacies, but rather develop the framework to fully represent them. Indeed, this is one of the main features of the Bayesian approach used here: having a probabilistic description of the results gives a natural way of describing indeterminacies and

multiple solutions to the inverse problem. We have added the following statement to introduction where the concept of indeterminacies are introduced:

"In this study, we focus on the latter problem of identifying parameter indeterminacies in a limited set of parameters, as to date there is no means to estimate large numbers of parameters at once. The goal is not to present a method to mitigate indeterminacies, but develop a framework that can fully represent them."

For example, in the last simulation considered, there is a clear overlap between drives to L2 neurons, EL2, IL", quantified by relatively large OVL values 0.190 and 0.011 (l. 655) and is left unattended.

The reason we introduce OVL is to allow a quantification of which parameters are separable under two different observations. Our goal isn't necessarily to minimize overlap, but provide a tool to identify which parameters you expect to be meaningfully different under different conditions.

l.391-401 The discussion about the utility of summary statistics could be motivated better or have parts removed. Of course, the most informative feature in a linear system would be the maximum number of principal components (PCA30 here, nothing surprising). Similarly, since observations are ERPs, it is expected that band power will be less informative.

The main goal of the first two examples is precisely to provide simple situations where we can anticipate the results. A primary reason for using band power as an example is that this is often used in neuroscience and Bayesian inference literature, particularly for MEG/EEG studies. See l.352 in the original manuscript:

"As we'll show below, this feature was intentionally selected as a cautionary example of a summary statistic that is ill-suited for the inference problem, but has a basis in previous neuroscience and Bayesian inference literature…"

Further, the examples shown in Figure 3 and 5 are not meant to be representative of an ERP, but rather could be a waveform shape of an oscillatory event that a researcher might naturally study using band-power. One of the take-home messages of our paper is to point out that one should use caution when inferring parameters that account for rhythmic activity in large scale neural models, and specifically that using band-power as a summary statistic may give indeterminate results.

Similarly, in the last example, only PCA30 results are considered (l. 640 and Figure 8) In general, what do we learn from the results about summary statistics besides that power is not an appropriate measure here? The authors should consider shortening the corresponding sections.

As discussed above, the point we are making with the summary statistic section is precisely that *power is not an appropriate measure*. We think this is an important point to make particularly for

the MEG/EEG community who often study rhythms and may want to use band-power as the summary statistic of interest. Of note, we only use PCA30 in Figure 8 because the goal of this figure is to exemplify how to infer and compare parameters from two different ERP waveforms. PCA30 was used because we showed above that this is a reliable summary statistic that captures the waveform shape.

We agree with the reviewer that the corresponding sections have some redundancy and can be shortened. To directly address this concern, we have removed the following sections that reiterate the same take-home message:

~~Defining gPRE as the global average value over this grid, the gPRE values for the parameter I_+ were low for \PCAthirty~(0.01 ± 0.02), \PCAfour~(0.03 ± 0.04), and \Peak~(0.04 ± 0.04) features. In contrast, the larger gPRE for Band Power features (0.23 ± 0.14) demonstrates that the ground truth for I_+ is recovered much less accurately. The gPRE for I_- followed a similar pattern, whereas the parameter Δt was generally well recovered for all ground truths (see \nameref{sec:data-code-availability} for results of diagnostics for all parameters and conditions).~~

~~Interestingly, the indeterminacy for the $\Delta t = 0$ waveform with \BandPower~features (\Cref{fig:hnn_diagnostics}\subfig{B(iv)}) is distinct from the RC circuit (\Cref{fig:rc_circuit_diagnostics}\subfig{B(iv)}) and exhibits a clear linear interaction.~~

~~Global PRE and PPC values confirm a similar ranking with \PCAthirty~performing best (P~-~gPRE: 0.005; gPPC: 8.675e-6\,nAm) and \BandPower~exhibiting higher values for both diagnostics (P~-~gPRE: 0.098; gPPC: 4.250e-5\,nAm) (see \nameref{sec:data-code-availability} for results of diagnostics for all parameters and conditions).~~

Also when talking about PCA , variance explained should be added in all instances this is used.

Thank you for the clarification. We have added the variance explained for PCA30 and PC4 after the first time they are mentioned in all of the examples.

l. 448 The simple linear RC circuit used as a first example has indeed some pedantic value. At the same time, it can be a bit misleading: the vast majority of interesting biophysical models are nonlinear, with much tighter posterior distributions, closer to the ground truth, like the one found here. In this case, interactions of the sort considered in the RC model are not of much practical relevance.

We definitely agree that the practical relevance of the RC circuit model is limited. However, while concepts such as structural non-identifiability are familiar to people with engineering/stats background, these sorts of examples are useful for neuroscientists (particularly MEG/EEG researchers) looking to use biophysical modeling in their work. Further, it’s much easier to understand how posterior diagnostics/checks should be used on simple examples where you can anticipate the results.

We respectfully disagree with the assertion that nonlinear biophysical models generally have tighter posterior distributions. This is highly dependent on the summary statistic and model parameters chosen. In this context of mapping macroscale waveforms to microscale model parameters, indeterminacies are generally expected to appear.

l. 613 Here and in other simulations only a few parameters are chosen: "parameters that define a small subset of local network …connectivity". How is then this approach dealing with high dimensional parameter spaces as it seems to be the claim?

Thank you for your comment! As stated above, our intention was not to suggest that this approach helps deal with a high dimensional parameter space. We felt that it was still important to mention that this is still one of the central difficulties of working with detailed models. As mentioned above we have now made explicit reference to this distinction in the introduction:

"In this study, we focus on the latter problem of identifying parameter indeterminacies in a limited set of parameters."

Discussion

l. 729 and below. Several parts need to be more accurate here. DCM does not employ a mean field assumption between parameters (see also remarks above regarding Introduction and Results) E-I balance has been discussed in several DCM papers, see e.g. Legon, W., Punzell, S., Dowlati, E., Adams, S. E., Stiles, A. B., & Moran, R. J. (2016). Altered prefrontal excitation/inhibition balance and prefrontal output: markers of aging in human memory networks. Cerebral Cortex, 26(11), 4315-4326. Also, Pinotsis, D. A., Perry, G., Litvak, V., Singh, K. D., & Friston, K. J. (2016). Intersubject variability and induced gamma in the visual cortex: DCM with empirical Bayes and neural fields. Human brain mapping, 37(12), 4597-4614.

We have removed the following section regarding the mean-field assumption:

~~*"For example, DCM employs the mean-field assumption, meaning that biologically important compensatory interactions such as excitatory/inhibitory (E/I) balance cannot be directly characterized in terms of synaptic conductance. Further, this means that DCM is not capable of representing the parameter indeterminacies with interactions that we showed can occur in the HNN-SBI framework."*~~

And have replaced it with the following text which highlights that the main benefit of SBI is flexibility in the choice of model and summary statistics (i.e. there is no need for an explicit likelihood function).

" However, their ability to make precise biophysical predictions on cellular and local circuit level processes is limited as the parameters are an abstraction representing population level activity~\cite{daunizeau_dynamic_2011}, preventing a one-to-one comparison between model

predictions and experimental measurements. Further, physiologically important effects like dendritic backpropagation are not represented in neural mass models unlike HNN."

Previous DCM work has used the HNN model, see Pinotsis, D. A., & Miller, E. K. (2020). Differences in visually induced MEG oscillations reflect differences in deep cortical layer activity. Communications biology, 3(1), 707, the authors present an approach for inferring parameters of detailed biophysical models based on statistical decision theory. It would be interesting to consider links between these approaches here.

Thank you for your comment. We have added the following discussion:

"Alternatively, previous works have attempted to merge the DCM and HNN modeling frameworks~\cite{pinotsis_linking_2017, pinotsis_statistical_2020}. This approach potentially enables one to benefit from the computationally efficient inference procedures offered by DCM, as well as the biophysical interpretability offered by HNN. Importantly, this approach necessitated 1) grouping of biophysical parameters in HNN to enable a one-to-one mapping to DCM parameters, and 2) an equivalent number of excitatory and inhibitory neurons in each cortical layer to permit a mean-field approximation and was applied to infer parameters in non-spiking HNN simulations of brain rhythms. For hypotheses where these assumptions apply and precise biophysical detail and network heterogeneity is not a focus the HNN-DCM approach offers a compelling alternative to HNN-SBI inference framework."

Another important development is the use of Bayesian Model Reduction that allows one to perform greedy search to compute model evidence in large spaces, see Friston, K., Parr, T., & Zeidman, P. (2018). Bayesian model reduction. arXiv preprint arXiv:1805.07092. This work should be discussed too.

Thank you for sharing this reference! We were unaware of this work but agree that BMR offers a principled approach to dealing with a large parameter space. We have added the following text to highlight this work:

"Another major limitation of the current framework is that a small number of parameters must be chosen to create a sufficiently large dataset for amortized inference. Recent methodological developments in Bayesian model reduction with DCM have shown that large parameter spaces can be searched over in a computationally efficient manner~\cite{friston_bayesian_2019}."

Minor comments

l. 169-182, l. 235-247 These sections seem more appropriate for the Methods section.

Since a primary message of the results is the influence of summary statistics on the results of SBI, we feel it is appropriate to keep these descriptions with the associated results sections.

l. 188 and below. It is a bit unclear what is step 1 vs 2 in Steps 1-2 etc. Better break down each step separately.

The second paragraph in this section corresponds to Step 2. We have broken them down as follows:

*“**Step 1: Define prior distribution.** SBI begins with…”*

*“**Step 2: Generate training data.** With the prior constructed…”*

l. 220 I guess here you refer to stochastic dynamics rendering ground truth parameters unknown. Better include a relevant discussion.

This is actually referring to the situation where face validity is being assessed, and the ground truth parameters are known for the simulated data. The sentence we’ve added to this section (in response to the face validity comment above) should clarify this confusion:

“...users can calculate the dispersion of the posterior around this ground truth using parameter recovery error (PRE). This is known as face validity when using simulated data to check if the ground truth parameters can be recovered \cite{kiebel_dynamic_2007, stephan_ten_2010}”

l.225 Is OVL more informative than other measures discussed (PRE, PPC)?

OVL is primarily informative when comparing inferred parameter distributions between two distinct observations. The central problem we are attempting to address is whether the model is able to capture distinct mechanisms underlying the differences in observed data. If we want to propose an experiment to test the predictions of our model, we see OVL as a tool to identify which parameters should be experimentally measured.

Fig3 and others. More detail is needed for Figure legends, e.g. there is no description for subpanels / e.g. various pair plots. Similarly for other Figure legends.

We have added the following text to the following figure captions;

Figure 3:

“\textbf{B}: Posterior distributions showing the inferred values that can generate the blue and orange waveforms from panel A (\PCAthirty~used to generate distributions) demonstrate that when the latency Δt between the inputs is zero, their amplitudes are indeterminate as visible as a highly dispersed distribution on panels B(a-c, blue), and with a positive correlation between the parameters I_+ and I_- on panel B(b, blue). In contrast, when $\Delta t \neq 0$ (orange), the distributions are tightly concentrated around the ground truth parameters (stars on panels B(a,c)) used to generate each simulation. The posterior distributions for the

parameter Δt are concentrated around the ground truth parameters for both conditions (panel B(f), blue and orange)."

Figure 5:

"\textbf{B}: Posterior distributions showing the inferred values that can generate the waveforms from panel A demonstrate that when the latency between the inputs is zero (blue), their amplitudes are indeterminate as visible as a dispersed distribution on panels B(a-c, blue), with a positive correlation between the parameters P and D on panel B(b, blue). Unlike the previous example (\Cref{fig:rc_circuit_schematic}), the indeterminacy is notably smaller for $\Delta t = 0$, with the posterior distributions primarily being concentrated around the ground truth parameters for P and D (stars on panels B(a,c))."

Figure 8:

"Overlap coefficients (OVL) quantifying the separability of the marginal posterior distributions conditioned on each waveform are shown on the diagonal for the corresponding parameters. $E_{\rm{L2}}$ and $I_{\rm{L2}}$ exhibit a small amount of overlap with OVL values of 0.011 and 0.190 respectively. In contrast $E_{\rm{L5}}$ and $I_{\rm{L5}}$ were much more distinguishable, exhibiting OVL values of 2.28e-5 and 1.59e-13 respectively."

l. 401 what are the "desirable properties" implied here is unclear to me.

We agree the phrasing here is a bit awkward. We have added the following text when this term is first introduced to clarify:

"Additionally, we introduce diagnostic analyses that can be used to quantitatively compare desirable properties of posterior approximations produced using different summary statistics, such as PPC, PRE, and OVL. Note that we use the term "desirable properties" as the goals of inference may differ depending on the use-case. For example, predicting precise values for biophysical values (parameter recovery) may be one goal. Alternatively, characterizing the range of parameters consistent with a known biomarker may be another goal."

l. 488-495 (around these lines) and l. 597-600 This could be more appropriate for the Discussion section.

We think these brief paragraphs of background information are important for motivating the examples in each respective section. We have attempted to make the indicated sections more concise as follows:

Beta Event description:

"Many studies have shown that Beta Events occur throughout the brain (e.g. see \cite{van_ede_neural_2018}) and their expression correlates with healthy and pathological

sensory and motor processing~\cite{shin_rate_2017, bonaiuto_laminar_2021, wessel_bursts_2020}. Further, they often have a stereotypical waveform shape that resembles an inverted Ricker wavelet lasting $\sim$150 ms~\cite{sherman_neural_2016, bonaiuto_laminar_2021, brady_periodicaperiodic_2022, wessel_-bursts_2020, cole_nonsinusoidal_2017}, see~\Cref{fig:beta_events}. HNN provides potential mechanistic explanations for how changes in waveform shape may emerge."

Event related potential description:

"HNN has also been applied to infer neural mechanisms underlying differences in ERP waveform shapes recorded across different experimental conditions (e.g, for tactile evoked responses in \cite{jones_neural_2007}). Here, we are not trying to reproduce any empirical findings but rather apply SBI to an ERP simulation as in \cite{jones_neural_2007} to examine the influence of changes in local network connectivity on the ERP waveform as a proof of concept example that examines a small subset of parameters distinct from our prior investigation."

l.564 -565 This seems to be an unnecessary repetition of the role of small distal variance in efficient inference.

This comment is no longer applicable as the Beta Event example section has been significantly modified in these revisions.

l. 593 please add "of" after "strength"

Fixed.

"The parameters regulating the timing and the strength of these drives…"

l. 649 please use plural: "differences" not "difference"

Fixed.

"...there are apparent differences in L5 pyramidal neuron spiking…"

l.787 the ending of the sentence sounds odd.

Agreed, we have replaced the sentence with:

"Further, significant research efforts currently underway have the potential to decrease the computational cost of likelihood-free inference, making these techniques more accessible."

Reviewer #2

The paper introduces the use of simulation based inference for Biophysically detailed neural modeling, that was developed by authors to study the multi-scale neural origin of human MEG/EEG signals, namely the Human Neocortical Neurosolver (HNN). Although the HNN and SBI method have been previously introduced and the novelty of the work is modest, the paper provides an excellent guide to inference in HNN. SBI approach is flexible and efficient method for Bayesian estimation of model parameters, and it is a wise choice for such models used in this paper.

The paper is well-written with clear structure. In particular, it is the strong point of the paper to investigate the relation between parameters for non-identifiability issue and the existence of degeneracy in model, with the diagnostics for reliability of the inference. This will be very useful to the community.

Thank you for the compliments!

Comments:

1) In abstract it would be clear to mention that the paper is focusing on simulated data, as a proof of concept. The SBI on empirical data will be more challenging.

In response to reviewer 1's comments we have now integrated an example with empirical data (the Beta Event example), along with a discussion on a limitation of this manuscript being a simplistic treatment of observation noise.

Introduction:

2) Following DCM, it is worth mentioning the new generation of automatic inference using HMC and its alternatives (ADVI/ MAPs) in probabilistic programming languages (PPLs such as Stan, PyMc3) for system neuroscience, especially for inference in epilepsy. Of course the challenges can be the efficient model's implementation in a specific language (in contrast to the flexibility of SBI), and differentiability due to the need for gradient calculation due to designed gradient-based algorithms in PPLs (NUTS/ADVI/MAP). In general, Bayesian inference using PPLs requires efficient reparameterization techniques as introduced by Jha et al, MLST 2022, doi: 10.1088/2632-2153/ac9037. Please also ee:

Hashemi, Meysam, et al. "The Bayesian Virtual Epileptic Patient: A probabilistic framework designed to infer the spatial map of epileptogenicity in a personalized

large-scale brain model of epilepsy spread." NeuroImage 217 (2020): 116839.

Hashemi, Meysam, et al. "On the influence of prior information evaluated by fully Bayesian criteria in a personalized whole-brain model of epilepsy spread." PLoS computational biology 17.7 (2021): e1009129.

Vattikonda, Anirudh N., et al. "Identifying spatio-temporal seizure propagation patterns in epilepsy using Bayesian inference." Communications biology 4.1 (2021): 1244.

Jha, Jayant, et al. "Fully Bayesian estimation of virtual brain parameters with self-tuning Hamiltonian Monte Carlo." Machine Learning: Science and Technology 3.3 (2022): 035016.

Thank you for these references! This is a very interesting line of inference techniques that we were not aware of while putting together this manuscript. We have added the following section to the discussion:

"*As an alternative to SBI, there has been substantial work in using probabilistic programming languages (PPL) to enable Bayesian inference on stochastic simulators. Models implemented in a PPL can be used in combination with modern inference algorithms (i.e. NUTS and ADVI) which have a dramatically lower computational cost compared to SBI. Unfortunately such approaches have the drawback that existing simulators must be completely rewritten to in a PPL which may even compromise the efficiency of running forward simulations. Nevertheless, these techniques have proved highly useful for inference in large-scale neural simulators, and can potentially be combined with surrogate models that approximate the forward simulations of existing models."*

3) line 57:
Hashemi et al. 2023 has investigated a reduced representation of neural dynamics at each brain region (slow-fast system), but coupled at whole brain level (even by 400 connected regions). The challenges and advantages of SBI (difficulty in data features calculation, large number of simulations, but capability to address degeneracy and amortized strategy for rapid hypothesis evaluation at patient-level) have been discussed in details.

We have amended this line as follows:

"...there is currently little guidance on how these methods should be used with large-scale biophysical models, with the notable exception of~\cite{hashemi_amortized_2023} offering a thorough discussion of using SBI on simplified models coupled in a large-scale brain network"

4) line 59: large-scale biophysical models;

Indeed, a situation that SBI is highly beneficial, is when the model is very high

dimensional (eg., whole brain models) but a few number of parameters is required to be estimated, for instance in Higgs Boson discovery, Cramer PNAS, and the following refs in system neuroscience for prediction on healthy aging and Alzheimer's Disease, respectively:

Lavanga, Mario, et al. "The virtual aging brain: a model-driven explanation for cognitive decline in older subjects." bioRxiv (2022): 2022-02.

Thank you for the references, the distinction of amortized/non-amortized inference is more thoroughly described in the discussion section so we have added the references there:

“In contrast, we omit the use of sequential methods to perform inference on multiple observations using the same trained neural density estimator, but at the expense of the number of parameters that can be inferred simultaneously. Nevertheless, when only a few parameters are necessary for estimation, SBI offers a powerful tool for amortized inference in high-dimensional models, with examples ranging from whole-brain models in systems neuroscience~\cite{lavanga_virtual_2022, hashemi_amortized_2023}, to particle physics models investigating the formation of the Higgs boson~\cite{brehmer_mining_2020}.”

5) Section HNN (after line 101):
Since the paper is mainly designed to infer the parameters of HNN, it is easier for the reader to get some details on the model eg in appendix, and what are the parameters (their dimension and their short description) as the target of estimation. I see Table 1, but it is worth a few lines to explain them in the main text. What is the dimension of equations, (missing) variables, and parameters to be estimated.

We have added the following section summarizing the parameters inferred in the main text:

“The results detail 3 examples of problems suited for parameter inference in HNN. In the first two examples, the parameters to be estimated control either the timing or strength of the input. In the first example (\Cref{fig:hnn_schematic}), the target of estimation is the parameter vector $\theta_0 \in \mathbb{R}^3$, where the first two parameters control the strength of a single spike of excitatory proximal and distal input (in units of nS for the synaptic conductance $\bar{g}$), and the third parameter Δt controls the timing between the two inputs (in units of milliseconds (ms)). In the second example \Cref{fig:beta_events}, the target of estimation is $\theta_0 \in \mathbb{R}^2$ where the parameters control the variance (ms^{2}) of a Gaussian distribution from which 10 spikes are drawn for the proximal and distal inputs. In the final example \Cref{fig:erps}, the target of estimation is $\theta_0 \in \mathbb{R}^4$, where the parameters control the synaptic strength (nS) of excitatory/inhibitory local connections onto the proximal/distal dendrites of L5 pyramidal neurons.”

6) Section Posterior diagnostics

Is PRE able to accurately quantify the error in the case of multimodality?

We are unsure if you are referring to multimodality in the sense of multiple recording modalities combined as a single observation, or in terms of the posterior distribution exhibiting multiple modes. In both cases, parameter recovery error (PRE) is well defined, as its only purpose is to measure the dispersion of the posterior around the ground truth parameters.

Introducing PPC as the root mean squared error can introduce serious issues to quantify the quality of fit and the predictions, especially for noisy time series. I do not expect to change the results, but at least it is expected to be pointed out in Discussion. The more principled way is to use rank plot, posterior shrinkages, z-score (to incorporeal the uncertainty in prior), and WAIC (see Hashemi etl., PLOS CB 2021, https://doi.org/10.1371/journal.pcbi.1009129).

Thank you for the reference! We agree that this is an overly simplistic introduction to PPC and have instead opted to include this comment in the section where PPC is first introduced instead of the discussion:

"We note that this is a simplified treatment of how to perform a PPC, as observation and neural noise may significantly change the interpretation of model fit. A more robust assessment of PPC in the context of systems neuroscience modeling can be found in~\cite{hashemi_influence_2021} which describes measures such as the Watanabe–Akaike information criterion (WAIC), as well as alternative measures to PRE for assessing posterior fit such as posterior shrinkages."

OVL is used to report the separability of posteriors across conditions? what is its advantage compared to KL or Ks distance?

That is correct, the only purpose of OVL in this manuscript is to quantify separability of posteriors. The main usefulness of OVL over KL or K's distance is pedagogic, by offering a readily interpretable answer to the question "how much do these distributions overlap?" on a scale of 0-1, which we think is a more natural question compared to "how far apart are these distributions?". We have added the following text to clarify this:

"OVL_k operates similarly to Kullback–Leibler divergence and related measures, with the primary advantage being that it is bounded on the interval [0,1] readily permitting identification of parameters with large and small amounts of distribution overlap."

7) Why the prior is transformed in the range of (0, 1)? Is this a sort of non-centered reparameterization? I did not get the motivation for transformation, and why for some linear/exponential.

Transforming the prior from the range of (0,1) is more of an implementation detail rather than a critical piece of information. Code-wise this makes the random sampling process more efficient, but is not important to include in the main text.

The linear/exponential transformation however is not an implementation detail, and is instead chosen to better reflect the dynamic range of these respective parameters. Notably for parameters controlling synapse strength, they tend to have a saturating impact on simulation outputs (increasing synapse strength past a certain threshold produces no appreciable differences in the model behavior). This has also been performed in previous work with SBI: "the synaptic conductances were transformed into logarithmic space before training and for the entire analysis".

"For parameters specifying the maximum conductance of synaptic connections in HNN, the values were exponentiated in base 10 after being mapped to the appropriate range. This is to account for the saturating impact of conductance on model outputs, and is employed in previous SBI work with biophysical models \cite{goncalves_training_2020}."

8) Page 7, section The important role of summary statistics:
As the data features play a critical role, what are the exact features for MEG used in this study? In Fig 3, only PCA30 or with statistical moments? In the later former case, how the mean, std of observation can be inferred from a mean of I_input with only PCA? also peaks of time series of the power spectrum of time series? It is more clear to mention dimension of each feature. How is the fitted data for this example. How the peaks are calculated? For noisy signal this can be challenging to define a peak and calculate data features. PCA30 is robust for signal to noise ratio?

Only the loadings of the principal components were used as the feature. We have added the following text to the relevant sections to clarify how PCA and "peak" summary statistics were implemented:

"In all subsequent examples, \PCAthirty{}~and \PCAfour{}~will refer to the $d=4$ and $d=30$ dimensional summary statistic vectors containing the loadings on the first 4 and 30 principle components. The PCA transformation was fit separately for each example using the dataset of 100,000 simulated time series waveforms (see \nameref{sec:supporting-information} for simulation details)."

"The first hand-crafted summary statistic we defined is a four dimensional vector including the amplitude and timing of the maximum and minimum peaks (\Peak) in the time domain of the simulated voltage response"

And this text to the discussion raising the point about sensitivity to noise:

"\PCAthirty was found to perform best for parameter recovery, but the information gained from low variance features may be highly sensitive to noise. Similarly, the hand-crafted summary

statistics like \Peak and \BandPower may be particularly impacted by noise.The sensitivity of neural density estimators to noise, and more generally identifying model misspecification, is an open problem in the SBI field \cite{ward_robust_2022, cranmer_frontier_2020} and will be an important direction for future research."

As mentioned in response to reviewer 1, a limitation of this study (and SBI in general) is a thorough treatment observation/neural noise in the inference procedure. This remains an open problem in the field and followup work will be necessary to understand the robustness of different summary statistics in the SBI framework.

A combination of all features will improve the inference compared to only PCA30?

A combination of all features will not necessarily improve inference. In the case of empirical data the model may have varying severities of model misspecification for different summary statistics.

9) The indeterminacies refers to the structural non-identifiability? (see https://doi.org/10.1016/j.neunet.2023.03.040 that distinguish structural and practical non-identifiability).

That is correct, in this setting we are primarily concerned with indeterminacies that arise from structural non-identifiability. Reviewer 1 has also pointed out that this manuscript is lacking discussion on sources of practical non-identifiability (i.e. modeling of neural vs. measurement noise). We have adopted the terminology of "indeterminacies" to stay consistent with the SBI literature this manuscript is based on. As noted above to comment 3, this reference has been highlighted in several places in the manuscript.

10) What is computational cost for the training and the dimension \theta, x_o. The cost of simulations can be addressed by parallel running on HPC, which is a great advantage of SBI compared to MCMC methods.

The computational cost for training is difficult to estimate as it depends on the convergence time. In general we find that NPE networks can be trained on a CPU in <1 hour for a dataset of 100,000 samples.

When distributed across 256 CPU cores, the simulated dataset for each example generally took <8 hours to generate. The neural density estimator took <20 minutes to converge when trained on the CPU of a single computing cluster node with 32 cores.

Typos:

subscription in GABAb, line 115, and caption of Fig 2, line 616, etc

We have updated the text to use $GABA_A$ and $GABA_B$ throughout the manuscript

in is repeated in line 607.

Fixed

"We start by simulating example ERPs with different peak amplitudes as shown ~~in~~…"
